# Microstructure and Self-Healing Capability of Artificial Skin Composites Using Biomimetic Fibers Containing a Healing Agent

**DOI:** 10.3390/polym15010190

**Published:** 2022-12-30

**Authors:** Qian Sun, Xu Gao, Sai Wang, Rong-Yue Shao, Xin-Yu Wang, Jun-Feng Su

**Affiliations:** 1 School of Information Engineering, Tianjin University of Commerce, Tianjin 300134, China; 2 School of Material Science and Engineering, Tiangong University, Tianjin 300387, China; 3 School of Mechanical Engineering, Tianjin University of Commerce, Tianjin 300134, China

**Keywords:** self-healing, artificial skin composite, fiber, healing agent

## Abstract

The aging and damage of artificial skin materials for artificial intelligence robots are technical problems that need to be solved urgently in their application. In this work, poly (vinylidene fluoride) (PVDF) fibers containing a liquid agent were fabricated directly as biomimetic microvasculars, which were mixed in a glycol–polyvinyl alcohol–gelatin network gel to form biomimetic self-healing artificial skin composites. The self-healing agent was a uniform-viscous buffer solution composed of phosphoric acid, acetic acid, and sodium carboxymethyl cellulose (CMC-Na), which was mixed under 40 °C. Microstructure analysis showed that the fiber surface was smooth and the diameter was uniform. SEM images of the fiber cross-sections showed that there were uniformly distributed voids. With the extension of time, there was no phenomenon of interface separation after the liquid agent diffused into the matrix through the fiber cavity. The entire process of self-healing was observed and determined including fiber breakage and the agent diffusion steps. XRD and FT–IR results indicated that the self-healing agent could enter the matrix material through fiber damage or release and it chemically reacted with the matrix material, thereby changing the chemical structure of the damaged matrix. Self-healing behavior analysis of the artificial skin indicated that its self-healing efficiency increased to an impressive 97.0% with the increase in temperature to 45 °C.

## 1. Introduction

As one of the most important organs of the human body, skin not only has the basic function of protecting the body, secreting oils, and excreting metabolites, but it is also an important system for human perception, interaction, and communication with the outside world [1,2]. Based on the functions of human skin, various artificial skins with corresponding functions have been developed. These bionics have extensive applications in body state detection, artificial limbs and robots, and human–computer interaction [3,4,5]. With the development of humanoid robots, more and more research has been carried out on artificial intelligence robot skin. With the gradual application of the artificial skin of robots in different fields, many problems have been revealed in the process, among which the breakage of artificial skin during use has become the primary problem that hinders the large-scale application of artificial skin. In practice, the broken area of artificial skin is small compared to the overall area, and replacing large areas of artificial skin can be costly financially and time-consuming [6,7,8].

It is well known that flexibility, tensile properties, and mechanical durability affect the service life of artificial skin [9]. Despite continuous refinement and improvement in the abovementioned directions, the problem of breakage repair in the use of artificial skin has never been well solved and has largely hindered its practical application [10,11,12]. Therefore, the ability of rapid repair after in-use damage is therefore key to the practical application of artificial skin. Self-healing technology has the advantages of low cost, long aging, easy preparation, and multiple materials selection. Nowadays, self-healing artificial skin has a broad application potential in the field of artificial limbs and artificial intelligent robots [13,14]. To prolong the service life of artificial skin, reversible-bond self-healing, microcapsule self-healing, and microvascular self-healing have been gradually explored [15,16,17,18].

Research shows that most reversibly bonded artificial skins, while solving the problem of self-healing, take a long time to repair after breakage and require a relatively demanding repair environment; these shortcomings limit the practical application of this class of artificial skin [19,20]. The appearance of microcapsule technology has enhanced the healing of microcracks in artificial skin and has enhanced the anti-aging effect of artificial skin. This technology enables it to achieve both anti-aging and self-healing. Nowadays, it is relatively mature, which has been proven in the fields of road and composite self-healing [21,22,23]. Reasonable design of the wall material can also make the core material continuously release to achieve the purpose of self-nutrition [24]. Low core content of microcapsules is less capable of repair in the face of larger damage to the artificial skin. Moreover, empty microcapsules tend to form stress concentrations after the core material is released, resulting in a reduction in the mechanical properties of the substrate [25]. Therefore, the direction of research to prolong the life of artificial skin should take account of self-healing properties and various mechanical properties [26].

In the face of the problems of reversible adhesive artificial skin in artificial intelligence robots prone to aging and microencapsulated artificial skin prone to stress concentrations due to a small amount of microcapsules, hollow fiber technology can be used not only as a reinforcement material to improve the mechanical properties of artificial skin, but also as a release healing agent to enable the artificial skin to self-heal after damage. This technology has been used successfully in the self-healing of asphalt, membranes, polymers, and other materials [27,28,29]. In previous work, self-healing artificial skin using fibers has been successfully designed with improved mechanical properties, which helps to promote the widespread application of artificial skin especially in artificial intelligence robot field [29,30,31]. A propylene glycol–polyvinyl alcohol–gelatin network gel is used as the matrix and polyvinylidene fluoride hollow fibers containing a healing agent are used as the reinforcement, with the fibers bonded to the gel matrix by being pre-laid into a mold. In previous studies, we have investigated the mechanical properties of artificial skin and the effect of the pH and the content of the restorative solution on the effectiveness of self-healing [32].

Based on the above research background and research basis, the microstructure and self-healing capability of artificial skin composites using biomimetic fibers containing a healing agent were investigated from the perspective of physics and chemistry. Microstructure analysis analyzed the fiber surface, distribution, and interface of this composite. The entire process of self-healing was observed and determined including fiber breakage and the agent diffusion steps. The self-healing agent release was detected based on the chemical reaction. Last, the temperature effect on self-healing efficiency was also investigated.

## 2. Materials and Methods

### 2.1. Materials

N, N-Dimethylacetimide (DMAC, ≥99.0%), poly (vinylidene fluoride) (PVDF), boric acid (≥99.5%), phosphoric acid (≥85 wt.%), an Acetate concentrated solution (≥99.8%), poly (vinyl alcohol) (PVA), gelatin (GEL, ≥99.5%), and glycerol (GL, ≥99.5%) were purchased from Aladdin Chemistry Co., Ltd., Shanghai, China. Sodium hydroxide (NaOH, ≥99.7%) and sodium carboxymethyl cellulose (CMC-Na) were purchased from Sinopharm Chemical Reagent Co., Ltd., Shanghai, China. All of the reagents were used as received.

### 2.2. Fabrication of Fibers Containing a Healing Agent

The fabrication method of PVDF fibers containing a healing agent has been reported in detail [30]. In this experiment, PVDF hollow fibers were prepared by wet–dry spinning [31]. The PVDF powder was dried in an oven (50 °C). The dried 100 g of PVDF powder and 400 g of DMAc solvent were placed in a sealed flask and stirred in a water bath (60 °C) for 8 h to obtain a casting solution. The casting solution was left to stand for 24 h under a vacuum of 0.1 MPa to remove air bubbles from the casting solution. The casting solution was transferred to a spinning tank at a temperature of 60 °C and the pressure source was nitrogen (0.15 MPa). At the same time, there was sufficient air in the core tank where the pressure value was maintained at 0.2 MPa. Under pressure, the casting solution and air were simultaneously injected into the spinneret head and spun in one step to form the hollow fiber’s structure. The tap water was used as an external coagulation bath kept at 25 °C. The hollow fiber containing an oily regenerant entered the outer coagulation bath and was wound onto the winding by means of a guide wheel. Ports of the individual hollow fiber were sealed with heat sealing scissors. Finally, the hollow fibers were removed from the winding and soaked in tap water at room temperature before use. The buffer solution used for the healing agent was 0.04 mol/L phosphoric acid and acetic acid. The pH of the buffer solution was controlled using NaOH solvent (0.2 mol/L) at 9.2%. Boric acid was added to the buffer solution and stirred to form a stable system. Finally, 0.5% CMC-Na was added to the system and the solution was stirred at 40 °C until a homogeneous viscous liquid was formed. The restorative solution was injected as a core liquid into the hollow fibers using a syringe and sealed with beeswax to prevent the restorative solution from escaping.

### 2.3. The Micro-Porous Structure of Bionic Fibers

The transverse cross-section and the shell of the bionic microvascular was observed by an SEM (Hitachi TM3030, Tokyo, Japan). The bionic microvascular was quenched in liquid nitrogen (−172 °C). Then, the bionic microvascular was adhered to one side of the sample stage using a conductive double-sided tape. The cross-section surfaces of the bionic microvascular were metal-sprayed, and morphologies were observed at an accelerating voltage of 30 kV.

### 2.4. Preparation of Artificial Skin Samples

The gel matrix of artificial skin is a typical network structure composed of PVA, GL, and GEL. Pour distilled water (35 mL) and GL (20 mL) were added into a beaker and heated in a water bath at 85 °C with a stirring speed of 500 r/min. Then, GEL (0.6 g) was added into the beaker and stirring continued at a speed of 1000 r/min for 30 min. This process can loosen the molecular chains of gelatin making GEL uniformly disperse into the gel system. When the solution was uniform and light yellow, PVA powder (5 g) was added into the beaker and the solution was heated to 100 °C with a stirring speed of 1200 r/min for 2 h. This process can break the intramolecular hydrogen bond of the PVA molecular chain on a microscopic level and form more intermolecular hydrogen bonds in the subsequent reaction. Finally, the bubbles were eliminated by an ultrasonic disperser.

The reaction mold consisted of two glass panes (2 mm × 8 cm × 8 cm), a silicone plate (3 mm × 8 cm × 8 cm), and three dumbbell-shaped grooves in the silicone plate with a length of 35 mm and a width of 15 mm. The glycol–polyvinyl alcohol–gelatin network gel in the solution was poured into the reaction mold; the fibers were pre-laid in the mold. It mold placed in a fridge (−24 °C) for 12 h and then moved to an oven (25 °C) for 2 h.

### 2.5. Microstructure Analysis

The transverse cross-section and the shell of fibers were observed by using a scanning electron microscope (SEM Phenom XL, Eindhoven, The Netherlands). The fibers were quenched in liquid nitrogen (−172 °C). Then, the fibers were adhered to one side of the sample stage using a conductive double-sided tape. The cross-section surfaces of the fibers were metal-sprayed, and the morphologies were observed at an accelerating voltage of 30 kV. Four artificial skin samples were prepared and sampled at the same locations of the four samples for 0, 6, 12, and 18 days. The crystallinity of the artificial skin and the gel matrix was measured by an X-ray diffractometer (XRD Germany BRUKER D8 DISCOVER) after freeze-drying. The scanning speed was 0.04°/s, and the diffraction angle (2θ) ranged from 5° to 40°.

Four artificial skin samples were prepared and sampled at the same locations in the four samples for 0, 6, 12, and 18 days and lyophilized and recorded by Fourier transform infrared spectroscopy (FT–IR Perkin Elmer Spectrometer 100) in the chemical band range of 400–4000 with a resolution of 4 cm^−1^.

### 2.6. Interface Morphology between the Gel Matrix and the Fibers

The morphology of the interface between the gel matrix and the fibers was observed by SEM (Phenom XL, The Netherlands and Regulus 8100, Tokyo, Japan). The artificial skin was quenched in liquid nitrogen (−172 °C). The samples were then glued to the side of the sample stage with conductive double-sided tape. The cross-sectional surface of the sample was sprayed with metal and the morphology was observed under an accelerating voltage of 30 kV.

### 2.7. Observation of the Diffusion of the Healing Agent in the Gel Matrix

The samples were artificial skin with a healing agent containing pH = 9 and 2.0% boric acid in the fiber and stained with methyl violet. The diffusion capacity was observed at 37 °C and photographed every 6 days using a camera (Canon EOS R6+_RF50mm F1.8 STM).

### 2.8. Measurement of the Self-Healing Capability of the Artificial Skin

The experiment was set up with three temperature control groups, one set of artificial skin and two sets of a gel matrix within each control group. A 5 mm wound was made with a scalpel on the artificial skin and one gel matrix within each control group, and the other group with a gel matrix was left untreated. They were left to stand for 24 h at 5 °C, 25 °C, and 45 °C. The tensile strength values of these samples were measured by using a universal testing machine (Shimadzu AGS-X 50 N, Kyoto, Japan) at a load of 50 N and a stretching rate of 1 mm/s at the corresponding temperatures. The formula for calculating the self-healing efficiency is as shown in the following Equation (1),

(1) $$P=\frac{\varepsilon_{a}}{\varepsilon_{b}}$$

where *P* is the healing efficiency, *ε_a_* is the artificial skin strain after self-healing, *ε_b_* is the artificial skin gel matrix strain.

## 3. Results and Discussion

### 3.1. Microstructure of Fibers

**Figure 1:**
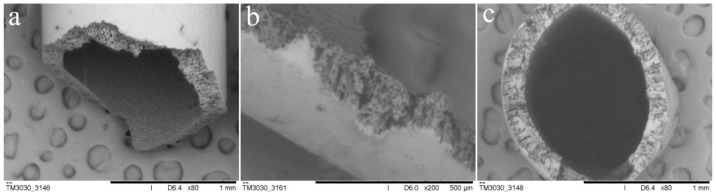
SEM cross-section morphology of hollow PVDF fibers: (**a**) the cross-section of a fiber with uniform thickness, (**b**) mall cavities in the fiber wall material with an average diameter of 1 μm, and (**c**) a fiber deformed after compression with a certain amount of mechanical strength.

Figure 1a–c shows the SEM cross-section morphology of hollow PVDF fibers from different angles of observation. From the figures, it can be roughly distinguished that the diameter of the fiber is 1 mm and the thickness of the fiber is 150 μm. The internal and external surfaces of the fibers are smooth without defects. The fiber cross-section presents a regular circle. It can also be seen from the shape of the cross-section that the fiber thickness is uniform without large deviation (Figure 1a). There are many small cavities in the fiber wall material with an average diameter of 1 μm, which is evenly distributed and has a uniform pore size (Figure 1b). The pore size is similar to the reported result [30]. Previous studies have also shown that these small cavities can allow the liquid inside the fiber to slowly release into the matrix material around the fiber [30,31]. The fiber has a certain mechanical strength and can be deformed after compression. From the shape of fiber compression (Figure 1c), it can be preliminarily determined that it can withstand a certain amount of pressure without damage.

### 3.2. Interface Morphology between the Gel Matrix and the Fibers

The interface problem of composite materials is a factor that cannot be neglected in materials research. The close bonding of the interface of each component in a composite material is the key to the overall performance of the composite material and the various materials that make it up. Therefore, for new composite materials, the interface stability between the gel and fibers of artificial skin is worth investigating. When the artificial skin is damaged with interface separation, the gel and fibers will not break simultaneously. However, the mechanical properties of the composite sample will be reduced along with the following two conditions. (A) The fibers break, and the gel does not break. The healing agent inside the fiber flows out and reacts with the gel, causing the healing agent to be wasted. (B) The gel is damaged, and the fibers are not broken. This situation is even worse than situation A and can leave the gel wound without timely healing and cause further wound enlargement during subsequent use of the artificial skin.

**Figure 2:**
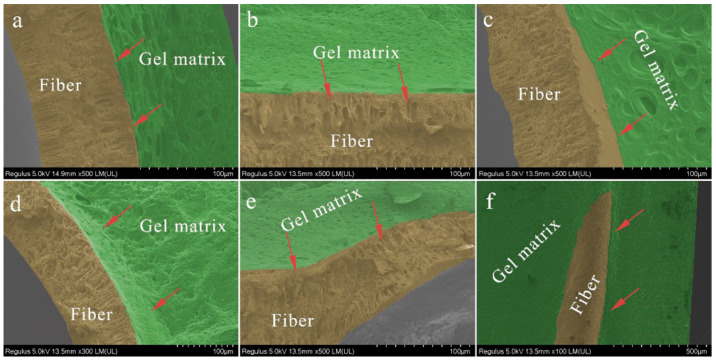
SEM morphology of interface morphology between the gel matrix and hollow fibers.

To address this vital issue, SEM was used to analyze the interface binding stability between the artificial skin fibers and gel. Interface damage is often very complex. In this study, the interface stability under steady-state conditions, stress damage, and material leakage are mainly considered. Figure 2 shows the interfacial bonding of the empty fiber and the gel after fracturing under different conditions. For better observation, we have post-colored these images, where the fiber part is colored yellow and the gel part is colored green. Figure 2a,b shows the SEM cross-section morphologies of an artificial skin sample observation from different angles. The cross-section is cut by freezing with a sharp slicer, and its surface is flat and smooth. The place pointed by the red arrow is the interface between the fiber and the gel. There is no apparent separation between the two interfaces, and the fracture surface is relatively neat and basically in the same plane. Therefore, it can be concluded that no two-phase interface separation between the fiber and the gel occurs in the face of tensile and shear forces exerted by sharp objects. Secondly, Figure 2c,d shows the artificial skin after it was stretched and fractured at the aperture. It can be noted that the fracture surfaces of the fiber and the gel are not at the same level, which is due to the different modulus of the two. Even so, the gel remains attached to the fibers, and it can be seen that even when the artificial skin is stretched with forces parallel to the interface, the gel remains firmly attached to the fibers without phase separation. Thirdly, Figure 2e,f shows the fracture of the artificial skin after fracture due to bending. It can be seen clearly that the fiber cross-section is damaged by repeated bending without phase separation between the fiber and the gel (Figure 2e). The gel remains intact. The artificial skin does not break after bending, but the gel appeared to fall off (Figure 2f). After careful observation, it can be clearly confirmed that the prominent part in the picture is the fiber, but the fiber surface is still wrapped by fiber. Therefore, it can be considered that the interface between the fiber and the gel is quite stable even after the fiber is bent.

**Figure 3:**
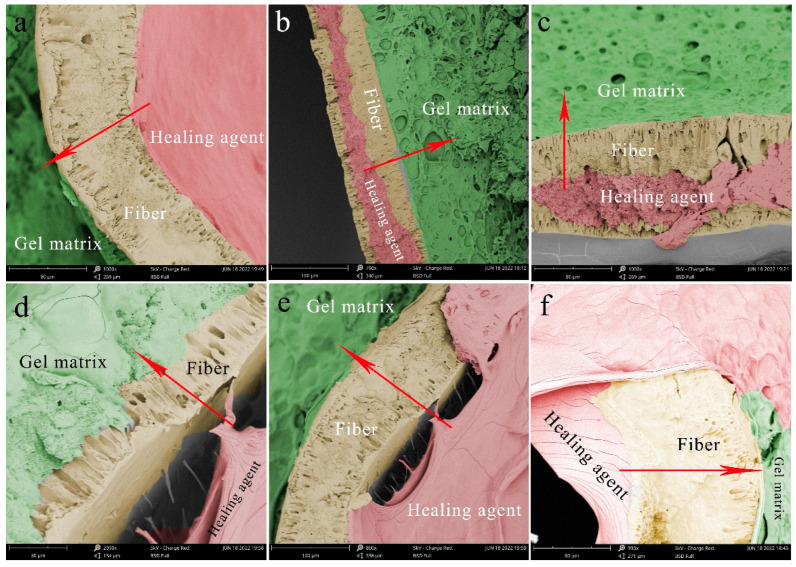
SEM morphology of interface morphology details between the gel matrix and fibers filled with a healing agent: (**a**–**c**) The healing agent penetrated the micro-porous structure. The red arrow indicates the direction of liquid penetration. (**d**–**f**) The whole process of liquid penetrating into the outer matrix of the fiber through micro-pores.

Figure 3 shows SEM images of the artificial skin after a period of self-healing and treatment breakage, in which the fibers are stained yellow, the gel is dyed green, and the healing agent inside the fibers is stained red. In Figure 3a–c, the healing agent has penetrated the micro-porous structure. The red arrow indicates the direction of liquid penetration. The micro-porous structure on the fiber wall in the figure was filled with the healing agent, and there was no phase separation between the gel and the fiber, indicating that the penetration of the healing agent did not affect the adhesion of the gel to the fiber. Figure 3d–f shows the whole process of liquid penetration into the outer matrix of the fiber through micro-pores. It can even be found that the healing agent infiltrates the gel through the micro-porous structure and fuses with the matrix gel. The reacted gel penetrates deeper into the fiber along the micro-porous structure, further strengthening the interfacial bond.

### 3.3. Determination of the Self-Healing Process

**Figure 4:**
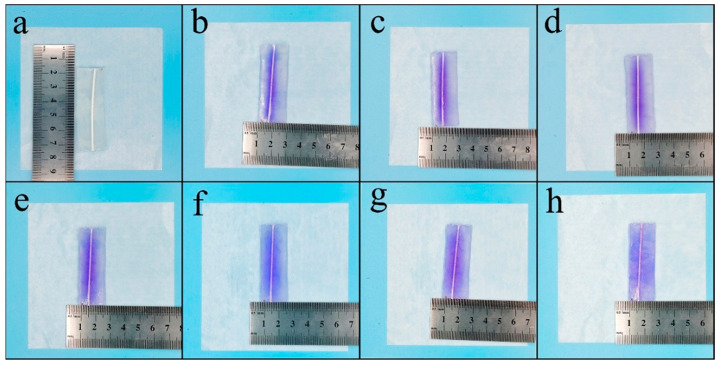
Photographs of the self-healing process with the agent diffusion process. (**a**–**d**) The healing agent spread to both sides with the fibers as the axis, and (**e**–**h**) the healing agent had spread throughout the gel by day 18.

There are numerous ways to identify self-healing, and direct observation is one of the most visual methods. The healing agent within the fibers in the artificial skin sample in Figure 4 was stained with methyl violet. The samples were left at 25 °C to observe the healing behavior of the healing agent on the gel, which was photographed at six-day intervals. As time progressed, the healing agent spread to both sides with the fibers as the axis (Figure 4a–d). By day 18, the healing agent had spread throughout the gel, and the color of the gel gradually deepened over the subsequent period (Figure 4e–h). There was no shrinkage in the gel size and no surface cracking during this period, indicating that self-healing of the gel by the fibers did occur.

**Figure 5:**
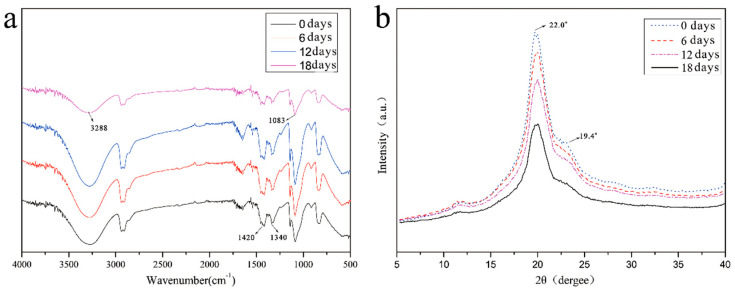
Chemical structures of artificial skin samples: (**a**) FT–IR curves of the exact location of the artificial skin on day 0, day 6, day 12, and day 18 of the self-healing process, and (**b**) XRD curves of the same location of the artificial skin on day 0, day 6, day 12, and day 18 of the self–healing process.

Figure 5a shows the FT–IR curves of the exact location of the artificial skin on day 0, day 6, day 12, and day 18 of the self-healing observation. These spectra have similar trends and peak positions; there are only minor differences in the peak position, intensity, and shape. The 3100–3700 cm^−1^ range has distinct peaks in all four spectra. Still, the peak intensity decreases sequentially with increasing self-healing time, which is characteristic of the O-H stretching vibration in the hydroxyl group, which reacts with boric acid to form borate ester bonds as the reflection proceeds. As the self-healing reaction proceeded, several distinct peaks of borates in the curve became more apparent, such as 1340 cm^−1^ and 1420 cm^−1^ (asymmetric stretching vibration peaks of the B-O-C complex). The characteristic peak of the spectrum at 1083 cm^−1^ was related to the vibrational stretching of the C-OH bond in the artificial skin and the matrix, and the C-OH content decreased with the self-healing reaction. The 1083 cm^−1^ characteristic peak also falls.

Figure 5b shows the XRD curves of the same location of the artificial skin on day 0, day 6, day 12, and day 18 of the self-healing observation. The curves show two distinct diffraction peaks at 2θ = 19.4° and 2θ = 22.0°, which correspond to the orthogonal lattice structure of the semi-crystalline PVA. With the self-healing of artificial skin, the diffraction peak at 2θ = 19.4° shifted and weakened. The diffraction peak at 2θ = 22.0° disappeared, indicating that the hydroxyl group of PVA reacted strongly with the borate, and the borate ester bond formed between them destroyed the structure of the crystalline region of PVA.

### 3.4. Measurement of the Mechanical Strength of the Composites at Different Temperatures

Temperature is a significant factor limiting the use of artificial skin. Temperature affects the mechanical properties of the artificial skin during regular use and its ability to heal itself. This experiment simulates the healing behavior of artificial skin at different temperatures including low temperature (5 °C), room temperature (25 °C), and high temperature (45 °C).

**Figure 6:**
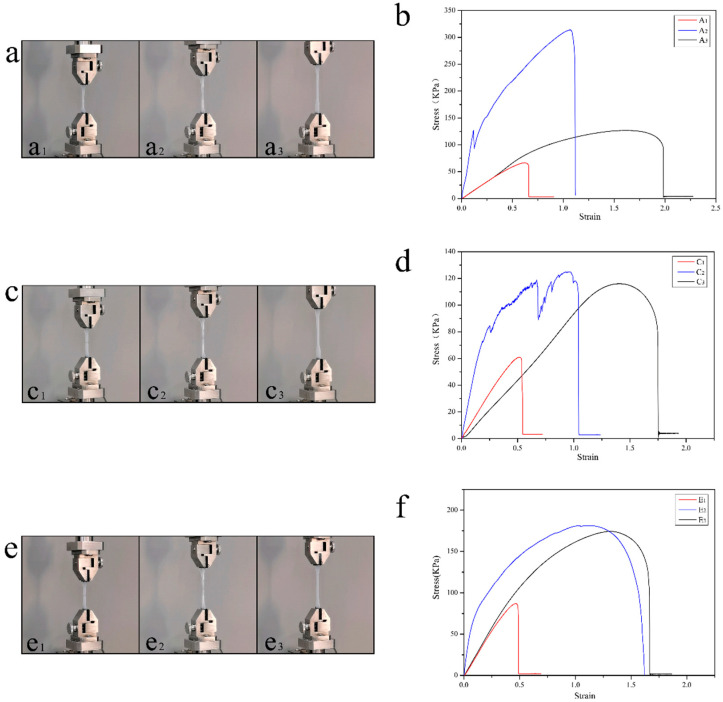
The artificial skin’s self-healing behavior at different temperatures. (**a**) The self-healing behavior of the artificial skin at 5 °C, (**a_1_**–**a_3_**) the damaged control group, the self-healing artificial skin, and the undamaged control group. (**b**) The stress–strain curves of artificial skin at 5 °C. (**c**) The self-healing behavior of the artificial skin at 25 °C and (**c_1_**–**c_3_**) the damaged control group, the self-healing artificial skin, and the undamaged control group. (**d**) The stress–strain curves of artificial skin at 25 °C. (**e**) The self-healing behavior of the artificial skin at 45 °C, (**e_1_**–**e_3_**) the damaged control group, the self-healing artificial skin, the undamaged control group. In addition (**f**), the stress–strain curves of the artificial skin at 45 °C.

Figure 6 shows a set of comparative experiments on the maximum stretching of artificial skin at 5 °C. Figure 6(a_1_) is the artificial skin without any treatment after destruction (named as A_1_), Figure 6(a_2_) is the artificial skin with self-healing after collapse (named as A_2_), and Figure 6(a_3_) is the artificial skin gel without any treatment (named as A_3_). It is evident that the maximum stretch of A_2_ is much larger than that of A_1_, and the stretch length of A_2_ is about 60% of that of A_3_. Figure 6b shows the stress–strain curves of the three groups of samples. Regarding strain, A_2_ (320 KPa) had much higher stress than A_3_ (127 KPa) during the stretching process. The lower temperature affected the performance of the artificial skin, making the performance of A_3_ lower, while the low temperature weakened the large elasticity of A_2_ while giving it greater strength. In terms of strain, the maximum strain of A_2_ was 1.2, the maximum strain of A_3_ was 1.96, and the repair efficiency of the artificial skin at 5 °C was 61.2%.

Figure 6c shows the comparative experiments of the maximum tensile degree of the artificial skin at an ambient temperature of 25 °C. The samples were treated the same way as the experiments at 5 °C named as C_1_, C_2_, and C_3_ as shown in Figure 6(c_1_–c_3_). Due to the increase in temperature, the difference between C_3_ (120 KPa) and C_2_ (130 KPa) in stress is insignificant. In terms of strain, the maximum strain was 1.3 for C_2_ and 1.75 for C_3_. The self-healing efficiency of the artificial skin at 25 °C was 74.3%, which shows that the increase in temperature enhanced the elasticity of the repaired artificial skin. However, it reduced the strength of the healed skin.

The ambient temperature of the comparison experiment of the maximum stretching degree of the artificial skin in Figure 6e is 45 °C, and the sample processing and experimental methods are the same as the previous two groups of experiments (Figure 6(e_1_–e_3_) are named E_1_, E_2_ and E_3_). Figure 6f shows the stress–strain curves of the three groups of samples. The stress of E_2_ (185 KPa) is slightly higher than that of E_3_ (175 KPa), the strain of E_2_ (1.65) is almost the same as that of E_3_ (1.7 KPa), and the repair efficiency reaches 97.0%. At higher temperatures, the self-healing reaction is more rapid and complete, so that the mechanical behavior of the repaired artificial skin can be restored to the previous level.

According to the performance of the artificial skin at different temperatures, the A_3_, B_3_, and C_3_ versions of artificial skin at various temperatures did not differ much, which is because the artificial skin gel matrix was prepared with the addition of propanetriol, a natural antifreeze agent, which ensured that the artificial skin maintained a more stable performance at different temperatures. The performance of temperature has a significant impact on the self-healing ability of the artificial skin. The temperature affects the self-healing reaction of the healing agent and the gel. Higher temperatures bring higher self-healing efficiency; at the same time, the temperature also affects the gel after the reaction. Higher temperatures result in a higher reaction degree, which indirectly improves efficiency.

## 4. Conclusions

The self-healing microvasculars can repair the skin of artificial intelligence ro-bots in times when the skin is damaged. At the same time, gland tubes in the skin can secrete oil, moisturize the skin, and delay skin aging. In this work, a self-healing glycol–PVC–gelatin network gel artificial skin was successfully fabricated composited with PVDF fibers containing a liquid agent. This self-healing artificial skin can realize self-healing and anti-aging functions, and it is expected to be applied to artificial intelligence robot skins to prolong the service life of the artificial skin and help it to better cope with various use scenarios. It was found that the artificial skin could achieve both self-healing and anti-aging functions, providing reduced cost and a new direction for the practicality of artificial skin. From the mentioned preliminary results, the following conclusions can be drawn:

(1) The microstructure of the fiber/artificial skin was studied by various methods in this study. It was found that the inner and outer surfaces of the fibers were smooth without defects. The cross-section of the fiber is a regular circle. There are many small holes in the fiber wall material with uniform distribution and pore diameter. After fracturing under different conditions, the hollow fiber and the gel are combined face-to-face. The observation results show that there is no phase separation between the fiber and the matrix material. The interface structure is stable and shall not be damaged.
(2) The self-healing process was determined including healing agent diffusion and crack healing. Firstly, the artificial skin samples were kept at 25 °C to observe the diffusion behavior of the healing agent into the gel, which was photographed at six-day intervals. As time progressed, the healing agent spread to both sides, with the fibers as the axis. Within 18 days, the healing agent had spread throughout the gel, and the color of the gel gradually deepened over the subsequent period. There was no shrinkage in the gel size and no surface cracking during this period, indicating that self-healing of the gel by the fibers did occur.
(3) The XRD and FT–IR results indicated that the self-healing agent could enter the matrix material through fiber damage or release and that it chemically reacted with the matrix material, thereby changing the chemical structure of the damaged matrix.
(4) Considering the tensile strength ratio before and after healing, the self-healing efficiency of the artificial skin composite was measured by a tensile fracture test. In order to simplify the complexity of the experiment, a single optical fiber was embedded in the matrix parallel to the tensile direction. It was found that the temperature greatly affected the self-healing efficiency.

## Data Availability

Not applicable.
